# Ergot of Rye a Remedy for Excessive Dilatation of the Pupil from Belladona

**Published:** 1848-11

**Authors:** 


*Ergot of rye a remedy for excessive dilatation of the pupil from Belladona.*—M. Comperat has announced a plan by which he has succeeded in removing dilatation of the pupil produced by belladonna in a patient of his, in whom the iris was scarcely visible, so complete had been the action of a small dose of belladonna applied externally. For some days the excessive dilatation resisted the employment of various collyria. He prescribed powdered ergot of rye, taken like snuff. The dilatation disappeared in a few seconds—it soon returned, the same remedy was again employed, and it did not reappear. He thought that ergot might be thus used in cases in which dilated pupil arises from other causes.—*London Med. Gaz.*

